# Clinical Impact of POU2F3 Expression in Surgically Resected Pulmonary High-Grade Neuroendocrine Carcinoma

**DOI:** 10.7759/cureus.82758

**Published:** 2025-04-21

**Authors:** Suguru Mitsui, Naoe Jimbo, Yugo Tanaka, Hiroyuki Ogawa, Shinya Tane, Daisuke Hokka, Yoshimasa Maniwa

**Affiliations:** 1 Thoracic Surgery, Kobe University Graduate School of Medicine, Kobe, JPN; 2 Diagnostic Pathology, Kobe University Hospital, Kobe, JPN; 3 Thoracic Surgery, Kobe University, Kobe, JPN

**Keywords:** high-grade neuroendocrine carcinoma, large-cell neuroendocrine carcinoma, pou2f3, small-cell lung cancer, transcription factors

## Abstract

Objective: High-grade neuroendocrine carcinoma (HGNEC) with dominant POU2F3 expression exhibits non-neuroendocrine features. However, clinical data regarding this subset of pulmonary HGNECs are scarce, and its clinical characteristics remain unclear.

Methods: Clinicopathological data from 109 patients who underwent surgery for HGNEC were collected and analyzed based on transcription factor expression. Patients were divided into a POU2F3-dominant group (HGNEC-P) and a non-dominant group (HGNEC-non-P) according to immunohistochemical analysis. The clinicopathological characteristics of the two groups were compared, and univariate and multivariate analyses were conducted to identify prognostic factors.

Results: The HGNEC-P group comprised 26 patients, while the HGNEC-non-P group comprised 83 patients. The HGNEC-P group showed significantly lower expression of carcinoembryonic antigen (CEA) (p < 0.001) and a lower rate of vascular invasion (p = 0.021) compared to the HGNEC-non-P group. In addition, the HGNEC-P group exhibited a unique tumor marker profile, with lower serum CEA and higher serum cytokeratin antigen (CYFRA) levels (p < 0.001 and p = 0.046, respectively). Complete resection was achieved in all HGNEC-P cases, whereas only 75.9% of HGNEC-non-P cases achieved complete resection. Multivariate analysis identified POU2F3 expression as an independent prognostic factor for recurrence-free survival (RFS) and disease-specific survival (DSS) (p = 0.037 and p = 0.038, respectively). In patients with pathological Stage I disease, the HGNEC-P group showed significantly better RFS (p = 0.010).

Conclusions: POU2F3-dominant HGNEC is associated with distinct clinicopathological features and favorable prognosis, particularly in early-stage disease. These findings may support the identification of this subset and inform the development of more effective treatment strategies.

## Introduction

High-grade neuroendocrine carcinomas (HGNECs), composed of cells with neuroendocrine (NE) characteristics, are classified as small-cell lung cancer (SCLC) and large-cell neuroendocrine carcinoma (LCNEC) in the lung. SCLC, accounting for approximately 13% of primary lung cancers, is known for its extremely poor prognosis [1]. LCNEC represents about 2-3% of primary lung cancers and is similarly associated with poor outcomes, comparable to those of SCLC [2]. While LCNEC shares a genomic profile with SCLC, it differs histopathologically and is characterized by a relatively high incidence of combined histologic features, observed in over 10% of cases [3]. Although the optimal systemic therapy for LCNEC remains controversial, recent studies have shown that treatment regimens similar to those used for SCLC may be more effective [4].

A recent molecular classification framework has categorized SCLC into four subtypes based on the expression of transcription factors: ASCL1, NEUROD1, POU2F3, and YAP1 [5]. SCLCs dominantly expressing ASCL1 or NEUROD1 typically exhibit high levels of thyroid transcription factor-1 (TTF-1) and NE markers, comprising the majority of SCLC cases. In contrast, SCLCs expressing POU2F3 or YAP1 show low levels of TTF-1 and NE markers [6,7]. While three subtypes (ASCL1, NEUROD1, and POU2F3) have been consistently demonstrated, a purely YAP1-positive subtype is not well established. The remaining category, lacking dominant expression of ASCL1, NEUROD1, and POU2F3, is referred to as inflammatory SCLC (SCLC-I) or triple-negative SCLC (SCLC-TN) [8-10].

Previous studies have indicated that each molecular subtype of SCLC has distinct biological and clinical characteristics, including variable responses to immunotherapy and molecularly targeted therapies [10-12]. However, only a few studies have explored this molecular classification in LCNEC. In our prior research, we found that both POU2F3-expressing SCLC and LCNEC shared morphological and phenotypical similarities, including near-negative expression of TTF-1 and carcinoembryonic antigen (CEA) on immunohistochemistry, and exhibited improved recurrence-free survival (RFS) compared to their non-POU2F3-dominant counterparts [6].

Given that POU2F3-dominant HGNEC exhibits unique features within the spectrum of NE carcinomas, we hypothesized that these tumors may also demonstrate distinct clinical characteristics and prognostic behavior. This retrospective study analyzes patients with surgically resected HGNEC to further investigate the clinical implications of POU2F3 expression, including multivariate survival analysis, and to support the development of more effective treatment strategies.

## Materials and methods

Study design

The study protocol was approved by the Clinical Research Ethics Committee of Kobe University Graduate School of Medicine (approval number: B230061). Informed consent was waived due to the retrospective nature of the study.

We collected information on the clinicopathological characteristics of patients (n = 112) who underwent pulmonary resection for HGNEC (including SCLC and LCNEC) between April 2001 and March 2023. The inclusion criteria were as follows: patients with medical details that could be obtained from medical records and those with pathological specimens. The exclusion criteria were as follows: missing data on physical examination, medical history, computed tomography (CT) scans, and pathological factors. The evaluation methods included physical examination, medical history, CT scans, brain magnetic resonance imaging (MRI), peripheral blood analyses, and pathological factors. Assessments of serum tumor marker levels, including CEA and cytokeratin antigen (CYFRA), were routinely performed within one month before surgery. Information on Tumor-Node-Metastasis (TNM) staging was based on the eighth edition of the TNM classification for lung cancer [13].

Morphology review and immunohistochemistry assessment

All pathological data were retrospectively reviewed. The histological diagnosis of SCLC and LCNEC was based on the fifth World Health Organization classification of tumors [14]. Pathological tumor factors, including histology, lymph node metastasis, and lymphatic, vascular, and pleural invasions, were evaluated. In this study, 11 types of antibodies, TTF1, Ki-67, classical NE markers including synaptophysin (SYN), chromogranin A (CGA), CD56, and INSM1, four novel transcriptional markers (ASCL1, NEUROD1, POU2F3, and YAP1), and CEA were used to examine protein expression using Ventana BenchMark GX (Roche, Basel, Switzerland), BOND-MAX/BOND-III (Leica, Deer Park, US), or the Dako Autostainer Link 48 (Agilent Technologies, Santa Clara, US) automated immunostainer.

Whole slides or spiral tissue arrays of formalin-fixed paraffin-embedded samples obtained from surgical specimens were used for immunohistochemical analyses. The expression of 11 markers was evaluated by the H-score. The H-score (0-300) is defined as the multiplication of intensity (0, 1 = weak, 2 = moderate, 3 = strong) and proportion (0-100%) of the expressed tumor cells according to a previously reported study [6,7,15]. For determining the dominant type in three transcriptional markers, including ASCL1, NEUROD1, and POU2F3, the marker showing the highest H-score of ≥50 was defined as the dominant type [6,7]. ASCL1 dominant, NEUROD1 dominant, and POU2F3-dominant groups were defined as the HGNEC-A, HGNEC-N, and HGNEC-P groups, respectively. The triple-negative group, in which three transcriptional marker scores were <50, was defined as the HGNEC triple-negative (HGNEC-TN) group. The HGNECC-A, HGNEC-N, and HGNEC-TN groups were defined as the HGNEC non-dominant (HGNEC-non-P) group. According to whether they were SCLC or LCNEC and POU2F3 expression, the SCLC-non-dominantly expressing POU2F3 (SCLC-non-P) group and the LCNEC non-dominantly expressing (LCNEC-non-P) group were defined.

Statistical analysis

Demographic characteristics of patients were expressed as the mean and standard deviation for continuous variables and as frequencies and proportions for categorical variables. RFS and disease-specific survival (DSS) were evaluated using the Kaplan-Meier method, and differences in survival curves were assessed using the log-rank test. RFS and DSS analyses were performed on residual tumor (R) 0 and R1 resection cases. Furthermore, univariate and multivariate Cox regression analyses of the associations between clinicopathological parameters and RFS and DSS were performed. Age, sex, pathological stage, chemotherapy, surgical procedure, and POU2F3 expression were used as covariates in the multivariate analysis. All statistical analyses were conducted using EZR version 1.55 (Saitama, Medical Center, Jichi Medical University, Saitama, Japan). EZR is a graphical user interface of R (The R Foundation for Statistical Computing, Vienna, Austria) [16]. A p-value of <0.05 was considered statistically significant. All p-values are two-sided and not adjusted for multiple testing.

## Results

Out of 112 patients, 109 met the selection criteria and were enrolled in this study. Three patients were excluded from the analysis: one had an unknown prognosis, and two had insufficient medical treatment information in their records.

Table 1 presents the association between POU2F3 expression and clinicopathological characteristics.

**Table 1:** Association between POU2F3 expression and clinicopathological characteristics Values are presented as n (%). Continuous variables are presented as the median and interquartile range (IQR). CEA: carcinoembryonic antigen; CYFRA: cytokeratin antigen; SCLC: small-cell lung cancer; LCNEC: large-cell neuroendocrine carcinoma; HGNEC-P: POU2F3-dominant high-grade neuroendocrine carcinoma; HGNEC-non-P: non-POU2F3-dominant high-grade neuroendocrine carcinoma

Factors		All patients	HGNEC-P group	HGNEC-non-P group	p-value
		n = 109	n = 26	n = 83	
Age	Median (IQR)	72.0 (66.0-77.0)	73.0 (70.3-76.8)	72.0 (65.0-76.5)	P = 0.276
Sex	Male	95 (87.2)	24 (92.3)	71 (85.5)	P = 0.511
	Female	14 (12.8)	2 (7.7)	12 (14.5)	
Smoking history	Yes	106 (97.2)	26 (100)	80 (96.4)	P = 1.000
Surgical procedure	Lobectomy or more	72 (66.1)	20 (76.9)	52 (62.7)	P = 0.237
	Sublobar resection	37 (33.9)	6 (23.1)	31 (37.3)	
Chemotherapy	Yes	55 (50.5)	13 (50)	42 (50.6)	P = 1.000
Radiotherapy	Yes	9 (8.3)	0	9 (10.8)	P = 0.194
Tumor size (mm)	Median (IQR)	25.0 (18.8-36.0)	27.0 (17.0-41.0)	25.0 (19.0-35.0)	P = 0.550
CEA (ng/mL)	Median (IQR)	4.6 (2.8-7.0)	2.9 (2.2-4.6)	5.3 (3.2-8.2)	P < 0.001
CYFRA (ng/mL)	Median (IQR)	1.3 (1.0-1.9)	1.6 (1.0-2.5)	1.2 (1.0-1.8)	P = 0.046
Histology	SCLC	70 (64.2)	17 (65.4)	53 (63.9)	P = 1.000
	LCNEC	39 (35.8)	9 (34.6)	30 (36.1)	
Pathological stage	I	51 (46.8)	14 (53.8)	37 (44.6)	P = 0.259
	II	32 (29.6)	6 (23.1)	26 (31.3)	
	III	18 (16.7)	6 (23.1)	12 (14.5)	
	IV	8 (7.4)	0	8 (9.6)	
Residual tumor classification	R0	89 (81.7)	26 (100)	63 (75.9)	P = 0.015
	R1	8 (7.4)	0	8 (9.6)	
	R2	12 (11)	0	12 (13.6)	
Lymphatic invasion	Positive	75 (69.4)	15 (57.7)	60 (72.3)	P = 0.225
Vascular invasion	Positive	89 (82.4)	17 (65.4)	72 (86.7)	P = 0.021
Pleural invasion	Positive	68 (63.0)	13 (50)	55 (66.3)	P = 0.166
Synaptophysin (H-score)	Median (IQR)	230 (30-300)	2.5 (0-17.5)	280 (200-300)	P < 0.001
Chromogranin A (H-score)	Median (IQR)	10 (0-80)	0 (0-0)	20 (0-100)	P < 0.001
CD56 (H-score)	Median (IQR)	270 (100-300)	110 (20-227.5)	290 (220-300)	P < 0.001
TTF-1 (H-score)	Median (IQR)	80 (0-270)	0 (0-0)	230 (5-280)	P < 0.001
INSM (H-score)	Median (IQR)	130 (20-260)	15 (1.25-37.5)	200 (75-270)	P < 0.001
CEA (H-score)	Median (IQR)	0 (0-110)	0 (0-0)	30 (0-135)	P < 0.001
ASCL1 (H-score)	Median (IQR)	120 (0-220)	0 (0-0)	200 (70-250)	P < 0.001
NEUROD1 (H-score)	Median (IQR)	0 (0-30)	0 (0-0)	5 (0-60)	P < 0.001
POU2F3 (H-score)	Median (IQR)	0 (0-50)	195 (92.5-257.5)	0 (0-0)	P < 0.001
YAP1 (H-score)	Median (IQR)	20 (0-180)	195 (50-235)	10 (0-65)	P < 0.001

The HGNEC-P group consisted of 26 patients (24.9%), and the HGNEC-non-P group included 83 patients (76.1%). Forty-nine patients received a platinum-based chemotherapeutic drug, and 47 received combination treatment with either irinotecan or etoposide. Two patients received tegafur-uracil orally after surgery. In four cases, the chemotherapy regimen was unknown. There were 70 patients (64.2%) with SCLC and 39 (35.8%) with LCNEC. Although no significant differences were found between the two groups regarding histology, pathological stage, or lymphatic and pleural invasion, the HGNEC-P group had a significantly lower proportion of patients with vascular invasion. All patients in the HGNEC-P group achieved complete tumor resection, compared to 63 patients (75.9%) in the HGNEC-non-P group.

**Figure 1:**
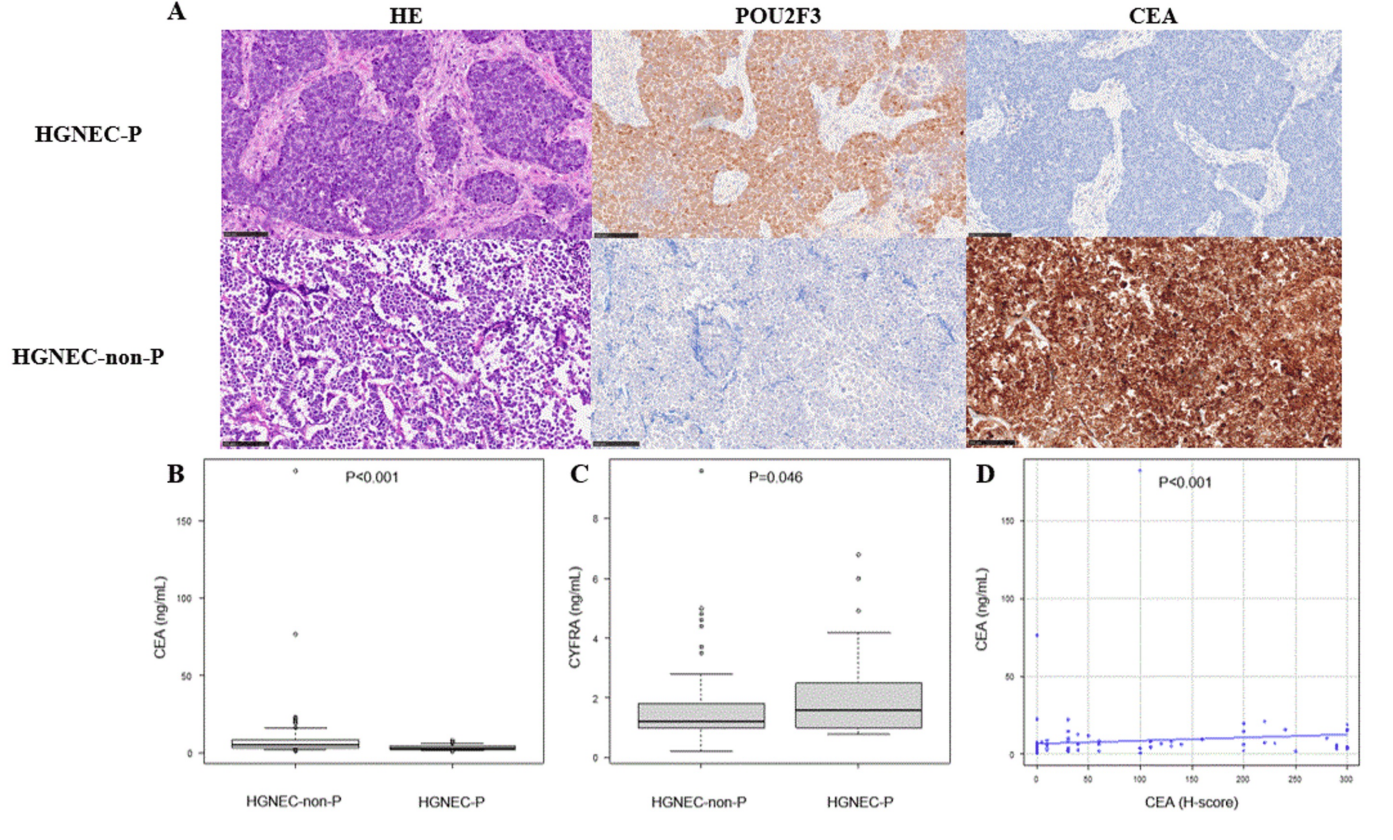
Immunohistochemical features and serum marker profiles according to POU2F3 expression status Staining of HE and immunochemistry in SCLC according to the POU2F3 expression status (A). There was no staining for CEA in the HGNEC-P group, as shown in a typical representative example. Serum CEA (B) and CYFRA (C) levels according to the POU2F3 expression status. Scatter plot showing that low CEA levels and high CYFRA levels were more likely associated with the POU2F3 expression status. A significant positive correlation was found between serum CEA levels and the H-score of CEA (D). HE: hematoxylin and eosin; CEA: carcinoembryonic antigen; CYFRA: cytokeratin antigen; SCLC: small-cell lung cancer; HGNEC-P: POU2F3-dominant high-grade neuroendocrine carcinoma; HGNEC-non-P: non-POU2F3-dominant high-grade neuroendocrine carcinoma

The HGNEC-P group also had significantly lower H-scores for NE markers, and no staining for CEA or TTF-1 was observed in this group, as shown in a representative case in Figure 1A.

Compared with the HGNEC-non-P group, the HGNEC-P group had significantly lower serum CEA levels and higher serum CYFRA levels (Figures 1B-1C). A significant positive correlation was also observed between serum CEA levels and the H-score of CEA expression (Figure 1D).

**Figure 2:**
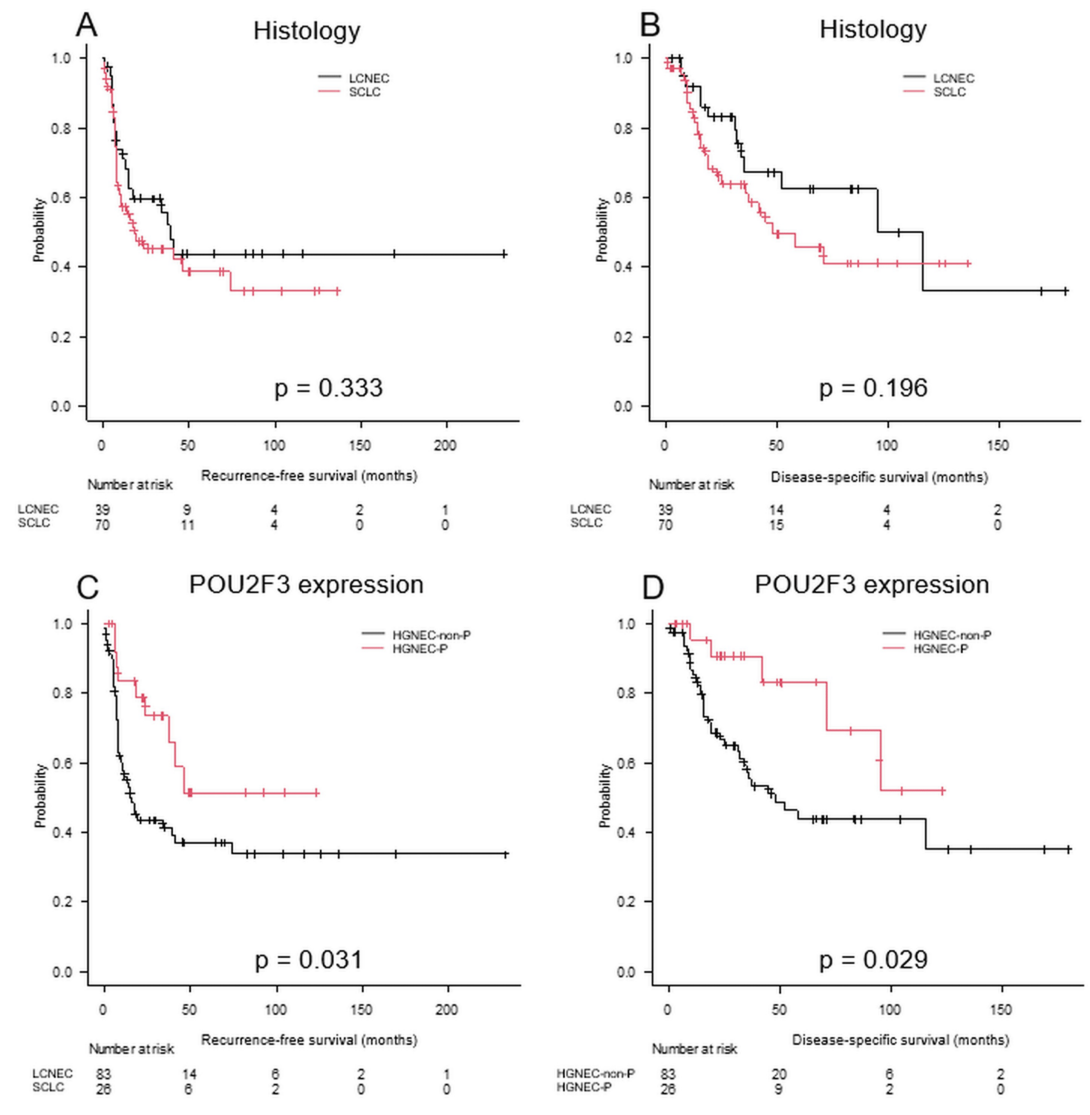
Survival analysis of resected HGNEC patients stratified by histology and POU2F3 expression RFS and DSS curves in patients with resected HGNEC based on histology (LCNEC vs. SCLC) (A, B), POU2F3 expression (C, D). The five-year RFS for the HGNEC-P and HGNEC-non-P groups were significantly different at 51.4% and 36.7%, respectively (p = 0.031). The five-year DSS rates for the HGNEC-P and HGNEC-non-P groups were significantly different at 83.1% and 42.4%, respectively (p = 0.029). RFS: recurrence-free survival; DSS: disease-specific survival; SCLC: small-cell lung cancer; LCNEC: large-cell neuroendocrine carcinoma; HGNEC-P: POU2F3-dominant high-grade neuroendocrine carcinoma; HGNEC-non-P: non-POU2F3-dominant high-grade neuroendocrine carcinoma

Figure 2 illustrates survival curves based on histology (LCNEC vs. SCLC) and POU2F3 expression status (dominant vs. non-dominant).

As shown in Figure 2C, the five-year RFS rates were 51.4% for the HGNEC-P group and 37.2% for the HGNEC-non-P group (p = 0.031). Similarly, Figure 2D demonstrates five-year DSS rates of 83.1% and 42.4% for the HGNEC-P and HGNEC-non-P groups, respectively (p = 0.029).

**Figure 3:**
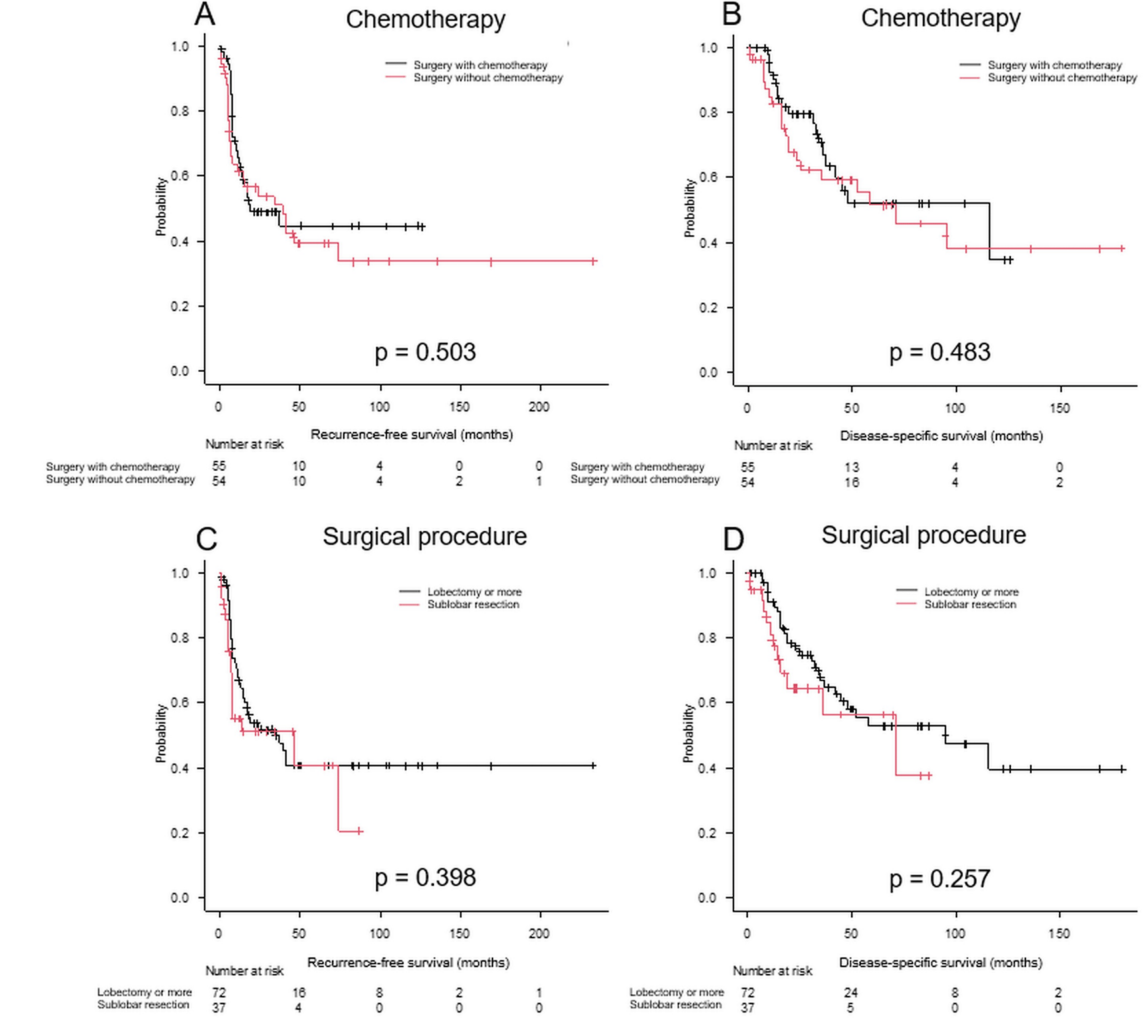
Survival analysis of resected HGNEC patients stratified by chemotherapy and surgical procedure RFS and DSS curves in patients with resected HGNEC based on chemotherapy (chemotherapy-treated vs. non-treated) (A, B), and surgical procedure (lobectomy or more vs. sub-lobar resection) (C, D). RFS: recurrence-free survival; DSS: disease-specific survival; HGNEC: high-grade neuroendocrine carcinoma

Figure 3 displays survival curves stratified by chemotherapy (treated vs. non-treated) and surgical procedure (lobectomy or greater vs. sublobar resection). No significant differences were observed in either comparison.

**Figure 4:**
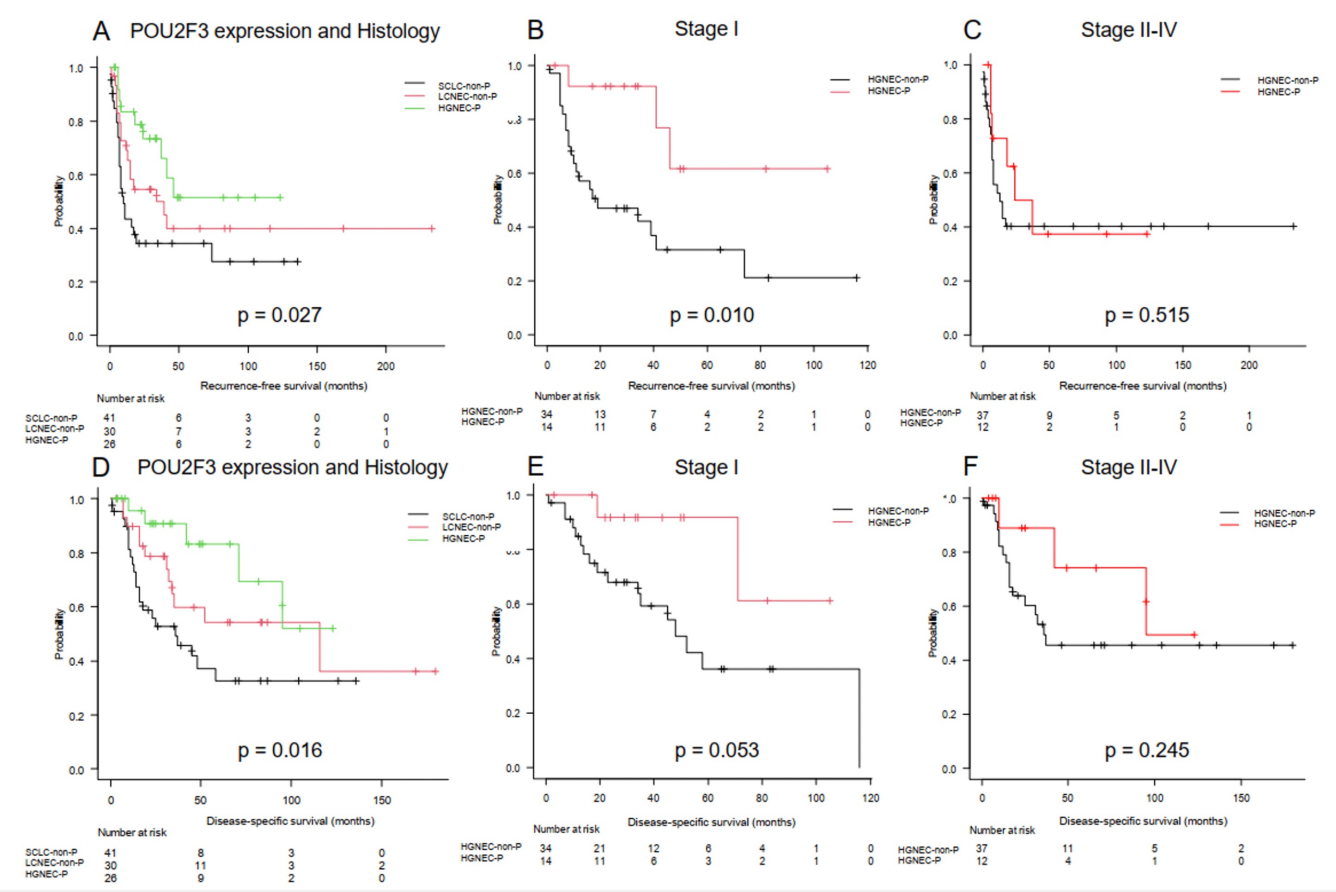
Survival analysis based on POU2F3 expression and histology in patients with resected HGNEC RFS curves in three-group comparison by POU2F3 expression and histology (HGENC-P vs. SCLC-non-P vs. LCNEC-non-P) (A). Figure 4A shows that the five-year RFS rates for the HGNEC-P, SCLC-non-P, and LCNEC-non-P groups were significantly different at 51.4%, 34.4%, and 40.0%, respectively (p = 0.029). RFS curves in patients with resected HGNEC based on POU2F3 expression in Stages I (B) and II-IV (C). Figure 4B shows that the five-year RFS rates in the stage I group of the HGNEC-P and HGNEC-non-P groups were 61.5% and 31.6%, respectively (p = 0.010). DSS curves in three-group comparison by POU2F3 expression and histology (HGENC-P vs. SCLC-non-P vs. LCNEC-non-P) (D). DSS curves in patients with resected HGNEC based on POU2F3 expression in stages I (E) and II-IV (F). Figure 4D shows that the five-year RFS rates for the HGNEC-P, SCLC-non-P, and LCNEC-non-P groups were significantly different at 83.1%, 32.5%, and 54.3%, respectively (p = 0.016). RFS: recurrence-free survival; DSS: disease-specific survival; SCLC: small-cell lung cancer; LCNEC: large-cell neuroendocrine carcinoma; HGNEC-P: POU2F3-dominant high-grade neuroendocrine carcinoma; HGNEC-non-P: non-POU2F3-dominant high-grade neuroendocrine carcinoma

Figure 4 shows subgroup analyses: a three-group comparison based on POU2F3 expression and histology (HGNEC-P, SCLC-non-P, LCNEC-non-P), and survival curves stratified by stage (Stage I vs. Stages II-IV) and POU2F3 expression.

The comparison among HGNEC-P, SCLC-non-P, and LCNEC-non-P revealed significant differences in RFS and DSS (p = 0.027 and p = 0.016, respectively), as shown in Figures 4A-4D. In patients with pathological Stage I disease, the HGNEC-P group showed a significantly better five-year RFS than the HGNEC-non-P group (p = 0.010, Figure 4B).

Table 2 summarizes the results of univariate and multivariate analyses for covariates associated with RFS and DSS.

**Table 2:** Univariate and multivariate analyses of covariables associated with recurrence-free survival and disease-specific survival HR: hazard ratio; CI: confidence interval

Outcomes (recurrence-free survival)		Univariate			Multivariate	
	HR	95% CI	p-value	HR	95% CI	p-value
Age (<75 vs. ≥75)	1.237	0.6654-2.2980	0.5019	1.8560	0.9233-3.7300	0.0826
Sex (male vs. female)	2.782	0.8634-8.9660	0.0866	3.6250	1.0920-12.040	0.0354
Pathological stage (I vs. II-IV)	1.262	0.7234-2.2030	0.4121	1.5850	0.8550-2.9370	0.4137
Perioperative chemotherapy (chemotherapy-treated vs. non-treated)	0.781	0.4458-1.3690	0.3882	0.6884	0.3729-1.2710	0.2325
Surgical procedure (lobectomy or more vs. sub-lobar resection)	0.6495	0.3540-1.192	0.1635	0.4729	0.2393-0.9344	0.0311
POU2F3 (dominant vs. nondominant)	0.462	0.2240-0.9508	0.0360	0.4606	0.2225-0.9537	0.0368
Outcomes (disease-specific survival)		Univariate			Multivariate	
	HR	95% CI	p-value	HR	95% CI	p-value
Age (<75 vs. ≥75)	1.206	0.5867-2.4810	0.6099	1.8260	0.8077-4.1260	0.1480
Sex (male vs. female)	2.209	0.6766-7.2120	0.1893	2.7890	0.8200-9.4860	0.1006
Pathological stage (I vs. II-IV)	1.024	0.5421-1.9350	0.9413	1.2230	0.6133-2.4380	0.5676
Perioperative chemotherapy (chemotherapy-treated vs. non-treated)	0.824	0.4382-1.5490	0.5479	0.7557	0.373-1.5100	0.4277
Surgical procedure (lobectomy or more vs. sublobar resection)	0.543	0.2717-1.0850	0.0838	0.4431	0.2063-0.9513	0.0368
POU2F3 (dominant vs. nondominant)	0.355	0.1386-0.9087	0.0308	0.3669	0.1425-0.9445	0.0377

In univariate Cox proportional hazards analysis, dominant POU2F3 expression was identified as a significant favorable prognostic factor. In multivariate analysis, POU2F3 expression remained an independent predictor for both RFS and DSS (p = 0.037 and p = 0.038, respectively).

## Discussion

In this retrospective study, the POU2F3-dominant group of HGNEC demonstrated a distinctive clinical and pathological profile. These tumors exhibited a unique serum tumor marker expression pattern (low CEA, high CYFRA), reduced vascular invasion, a higher rate of complete surgical resection, and better disease control in pathological stage I compared to the HGNEC-non-dominant group. Comparison among three subgroups - HGNEC-P, SCLC-non-P, and LCNEC-non-P - revealed significant differences in both RFS and DSS. Moreover, multivariate analysis identified POU2F3 expression as an independent prognostic factor for RFS and DSS, suggesting that patients with POU2F3-dominant HGNEC may benefit from more active treatment strategies.

Among the four transcription factor subtypes proposed for SCLC - ASCL1, NEUROD1, POU2F3, and YAP1-POU2F3 - are mutually exclusive with ASCL1 and NEUROD1, and lack typical NE features [5,7]. POU2F3 is a master regulator critical for tuft cell development, a chemosensory lineage observed in small populations within the tongue, respiratory tract, intestine, and other epithelial tissues [12,17,18]. The categorization of YAP1 as an independent SCLC subtype remains controversial, as YAP1-positive cases may reflect contamination from non-SCLC. The present study was based on our previous classification, which defined the fourth transcriptional type as triple-negative (TN) [6]. However, Gay et al. proposed an alternative classification that identified an inflammatory gene signature as the fourth subtype [8].

A previous retrospective study reported that POU2F3- and YAP1-positive subtypes, both lacking NE features, were associated with favorable outcomes, with three-year overall survival (OS) rates of 68.9% and 82.4%, respectively [17]. In our prior study, the five-year OS rate was 43.8% in POU2F3-dominant SCLC and 75.2% in POU2F3-dominant LCNEC [6]. These outcomes may be attributed to the high rate of complete resection and the low incidence of vascular invasion in the POU2F3-dominant group, suggesting reduced local invasiveness. In contrast, there was no significant difference in prognosis between the POU2F3-dominant and non-dominant groups among patients with Stages II-IV tumors, suggesting that local control may be difficult in advanced HGNEC regardless of subtype. The finding that prognosis significantly differs among the three groups (HGNEC-P, SCLC-non-P, and LCNEC-non-P) is novel and noteworthy.

Tumors expressing POU2F3 are considered tuft-cell-like carcinomas and exhibit a range of histologic types beyond HGNEC. These tumors often share transcriptional profiles characterized by overexpression of BCL2, KIT, and MYC [19]. In the same study, although the clinicopathologic features of tuft-like NEC and tuft-like squamous cell carcinoma (SQ) were different, both tumor types co-expressed BCL2 and KIT. In our previous report, POU2F3-dominant HGNEC frequently showed combined NEC with SQ and higher expression of p40 and CK5/6 [6]. The elevated CYFRA levels observed in the POU2F3-dominant group may reflect the shared molecular features of tuft-like NEC and tuft-like SQ, which may derive from the same differentiation lineage. The lower serum CEA levels are consistent with the immunohistochemical findings and support the hypothesis that POU2F3-dominant HGNEC may originate from a non-NE lineage. Thus, these two tumor markers may be clinically useful in selecting patients with POU2F3-dominant HGNEC, particularly during biopsy-based diagnosis.

In recent years, personalized treatment approaches for lung NE carcinoma have been increasingly investigated [10-12]. NEUROD1-dominant SCLC has been shown to respond to aurora kinase inhibitors [11], while preclinical studies have suggested that POU2F3-dominant tumors may be sensitive to IGF-1R inhibitors, PARP inhibitors, and lurbinectedin [8,12]. The CASPIAN trial, a randomized study of durvalumab (with or without tremelimumab) plus platinum-etoposide in treatment-naive patients with extensive-stage SCLC, demonstrated that treatment efficacy varied according to transcription factor subtype [20]. While PD-1 inhibitors may be beneficial in the inflammatory subtype [17], their effect in POU2F3-dominant HGNEC remains unclear due to the small number of patients studied. Notably, our analysis excluded patients who received immunotherapy, and no difference in response to platinum-based chemotherapy was observed between POU2F3-positive and negative groups. Considering the high complete resection rate and relatively favorable prognosis in Stage I, aggressive treatment may be more effective in POU2F3-dominant SCLC and LCNEC than in typical NE subtypes.

This study has several limitations. First, it was a retrospective analysis conducted at a single institution. Second, molecular subtyping was based solely on immunohistochemistry, and gene-level differences were not explored. The method for determining dominant transcription factor expression is not standardized, and cutoffs such as the H-score ≥50 may vary by study. Third, only 50% of patients received chemotherapy, and only 8% received postoperative radiotherapy. Finally, as with all retrospective studies, unmeasured confounding and information bias may exist.

## Conclusions

This analysis of transcription factor-defined subgroups in HGNEC reveals several key findings. First, POU2F3-dominant HGNEC exhibits distinct clinicopathologic characteristics, including low serum CEA, high CYFRA levels, reduced vascular invasion, and favorable prognosis in pathological Stage I, likely owing to a higher rate of complete surgical resection. Second, POU2F3 expression was identified as an independent prognostic factor for both RFS and DSS, supporting its role as a biologically relevant marker for risk stratification. Third, POU2F3-dominant HGNEC displays a non-NE immunophenotype and may originate from tuft cell-like lineage, suggesting potential diagnostic and therapeutic implications, particularly in biopsy-based settings. These findings may help in selecting patients who are more likely to benefit from aggressive local treatment and in identifying new therapeutic targets for this distinct subgroup.
